# Synthesis, identification and in vivo studies of tumor-targeting agent peptide doxorubicin (PDOX) to treat peritoneal carcinomatosis of gastric cancer with similar efficacy but reduced toxicity

**DOI:** 10.1186/1476-4598-13-44

**Published:** 2014-03-03

**Authors:** Li Tang, Rui Duan, Yan-jun Zhong, Raymond A Firestone, Ya-ping Hong, Ji-guo Li, Yan-chao Xin, Han-lin Wu, Yan Li

**Affiliations:** 1 Department of Oncology, Zhongnan Hospital of Wuhan University, Hubei Key Laboratory of Tumor Biological Behaviors & Hubei Cancer Clinical Study Center, No 169, Donghu Road, Wuhan 430071, China; 2 Department of General Surgery, Jingmen First People’s Hospital, Jingmen 448000 Hubei, China; 3 Nanjing Meihua Pharmaceuticals, Ltd, Nanjing 210009, P. R. China; 4 Princeton Global Synthesis LLC, 360 George Patterson Blvd. Suite 206, Bristol, PA 19007, USA

**Keywords:** Peritoneal carcinomatosis, Gastric cancer, Peptide doxorubicin, Cytoreductive surgery, Hyperthermic intraperitoneal chemotherapy

## Abstract

**Background:**

This work aimed to synthesize a cathepsin B (CTSB)-cleavable tumor-targeting prodrug peptide doxorubicin (PDOX) and study the in vivo efficacy and toxicities on an animal model of gastric peritoneal carcinomatosis (PC).

**Methods:**

PDOX was synthesized using doxorubicin (DOX) attaching to a CTSB-cleavable dipeptide Ac-Phe-Lys and a para-amino-benzyloxycarbonyl (PABC) spacer. PC model was established by injecting VX2 tumor cells into the gastric sub-mucosa of 40 rabbits, which then were randomized into 4 groups: the Control (n = 10) without treatment, the HIPEC (n = 10) receiving cytoreductive surgery (CRS) plus hyperthermic intraperitoneal chemotherapy (HIPEC), the PDOX (n = 10) and the DOX (n = 10) receiving systemic chemotherapy with PDOX 50.0 mg/kg or DOX 5.0 mg/kg, respectively, after CRS + HIPEC.

**Results:**

The median overall survivals (OS) were 23.0 d (95% CI: 19.9 d - 26.1 d) in the Control, 41.0 d (36.9 d - 45.1 d) in the HIPEC, 65.0 d (44.1 d - 71.9 d) in the PDOX, and 58.0 d (39.6 d - 54.4 d) in the DOX. Compared with the Control, the OS was extended by 70% in the HIPEC (*p* < 0.001) and further extended by 40% in the DOX (*p* = 0.029) and by 58% in the PDOX (*p =* 0.021), and the PC severity was decreased in the HIPEC and further decreased in the PDOX and DOX. Animals receiving DOX treatment showed hematological toxicities with marked reduction of white blood cells and platelets, as well as cardiac toxicities with significant increases in creatine kinase mb isoenzyme, evident myocardium coagulation necrosis, significant nuclear degeneration, peri-nucleus mitochondria deletion, mitochondria-pyknosis, and abnormal intercalated discs. But these toxicities were not evident in the PDOX.

**Conclusions:**

PDOX is a newly synthesized tumor-targeting prodrug of DOX. Compared with DOX, PDOX has similar efficacy but reduced hematological and cardiac toxicities in treating rabbit model of gastric PC.

## Background

GC is one of the most common malignancies in developing countries, where it ranks second in terms of incidence rate and third in terms of mortality rate among the male population, and ranks fourth in terms of both incidence rate and mortality rate among the female population, according to the most recent global statistics [1,2]. GC is also the third leading cause of cancer mortality in China [3], where over 70% of GC has already become clinically advanced by the time of surgical exploration, thus surgical resection alone is no longer curative [4].

PC in GC has long been considered as a fatal clinical entity. It is defined as the implantation of tumor cells throughout the peritoneal cavity and characterized by the presence of tumor nodules of various size, number, and distribution on the peritoneal surface as well as malignant ascites, frequently resulting in locoregional morbidity without broader systemic metastases. Patients with gastric PC face a dismal outcome, with a median survival of about 6 months [5].

There is no standard treatment for gastric PC. Current treatments for such PC are systemic chemotherapy, best support care and palliative therapy. In order to tackle this problem, a new treatment modality called CRS plus HIPEC has been developed over the past 3 decades, taking advantages of surgery to reduce visible tumor burden, and regional hyperthermic chemotherapy to eradicate micrometastases [6]. Increasing evidence has suggested that the combination of CRS and HIPEC could bring survival benefit for selected patients with gastric PC. In our previous experimental study [5], we have also proved that CRS + HIPEC could indeed bring survival benefit with acceptable safety, providing evidence to support this combined strategy to treat selected patients with gastric PC.

During the development of PC, GC cells secrete enzymes to facilitate cancer cells seeding and colonization on the peritoneum. CTSB is one of the key enzymes in this critical process, over-expressed in GC as well as other cancers [7-9] and actively involved in cancer invasion [10-12]. On the other hand, it is extremely low expressed in normal cells and inactive or loses activity as soon as it is dispersed in aqueous media away from cells [13]. Thus CTSB has long been considered as a candidate target in cancer therapy [14].

**Figure 1:**
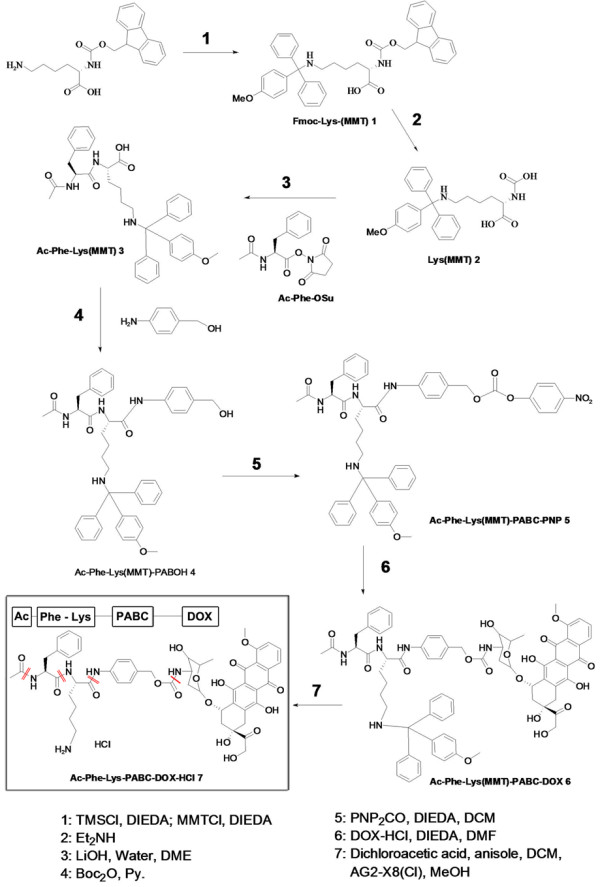
**PDOX structure (shown in the box), chemical synthesis and action mechanism.** PDOX was successfully synthesized according to the 7-step chemical process. Its chemical structure is Ac-Phe-Lys-PABC-Dox·HCl (shown in the box), and the molecular formula is C_52_H_60_ClN_5_O_16_. PDOX contains a CTSB-cleavable dipeptide Ac-Phe-Lys (double red slashes) a PABC spacer (red slash) and anti-cancer drug DOX. When PDOX reaches CTSB-enriched area such as the invasion front of cancer, the Ac-Phe-Lys dipeptide is cleaved by CTSB at the Lys-PABC bond, exposing the PABC spacer that is then hydrolyzed spontaneously (red slash), releasing free DOX at the cancer invasion front. Thus PDOX could exert cytotoxicity to invading cancer cells while protecting normal cells from excessive drug exposure, a strategy called passive targeted therapy. CTSB: cathepsin B.

It has been established in the MAGIC trial that the anthracycline-contained regimen is a useful chemotherapy for GC [15]. DOX is a typical representative of anthracyclines. Although DOX is an important drug in chemotherapy, its toxicities are evident, such as cardiac toxicities and bone marrow suppression. To retain the therapeutic effect while reducing the side effects, Dubowchik et al [16-18] designed a smart prodrug of DOX, PDOX (Figure 1). In this modified DOX, Ac-Phe-Lys is a dipeptide specific for CTSB, and PABC (para-aminobenzyloxycarbonyl) is a self-immolative spacer [16]. The prodrug is inactive when there is little CTSB activity, such as normal tissues and peripheral blood, thus avoiding the side effects on normal tissue. During cancer invasion, activated CTSB is over expressed on the exterior membrane of the invading cancer cells [19,20], which cleaves the Ac-Phe-Lys dipeptide at the Lys-PABC bond [16]. Then the exposed PABC spacer can self-hydrolyze upon deacylation [21] and free DOX molecules are released, resulting in direct killing of the invading cancer cells [16].

In this study, we synthesized the PDOX and evaluated the efficacy and safety of CRS + HIPEC with molecular targeted therapeutic regimen PDOX for targeted treatment of rabbit model of gastric PC.

## Results

### Synthesis and identification of PDOX

PDOX was successfully synthesized according to the previously reported 7-step chemical process (Figure 1) [16-18].

**Figure 2:**
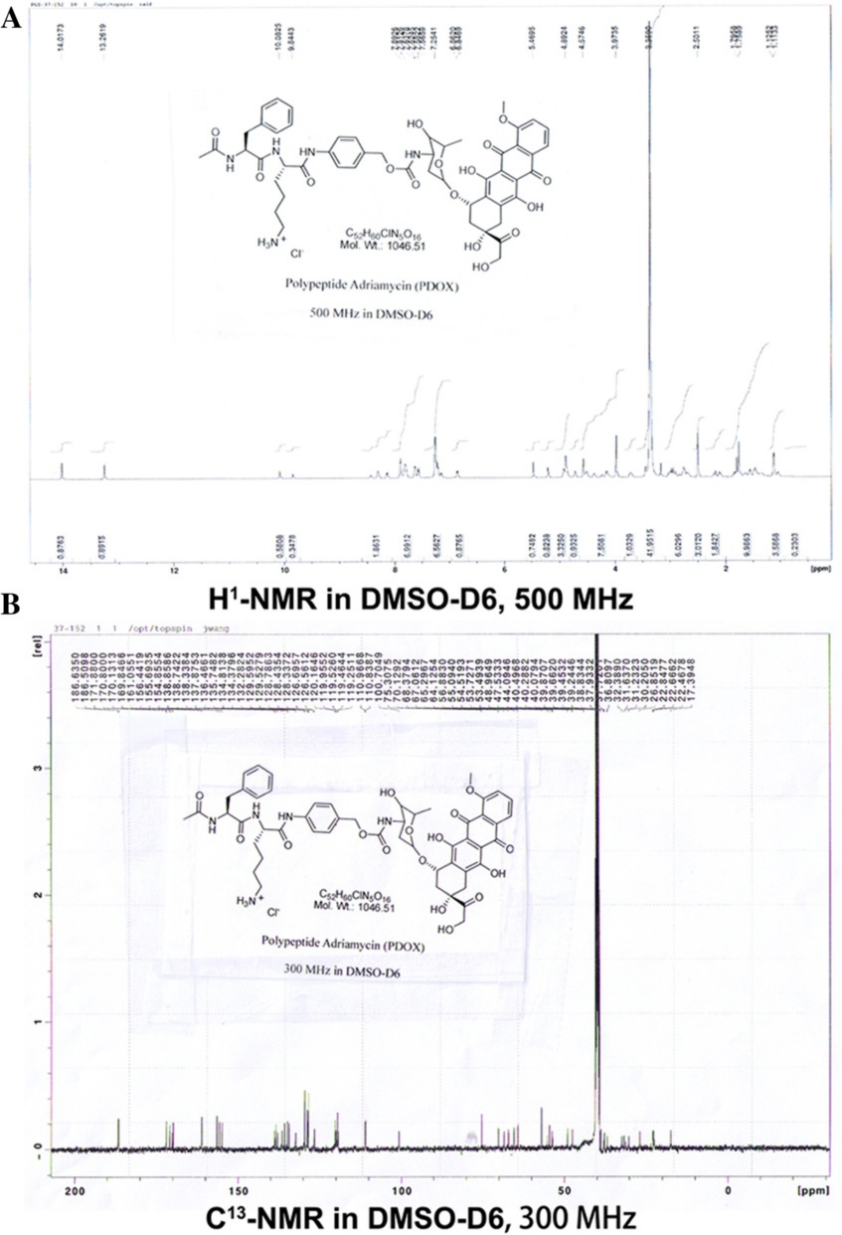
**Structure identifications confirmed by H**^**1**^**-NMR (A) and C**^**13**^**-NMR (B).**

PDOX (Ac-Phe-Lys-PABC-Dox · HCl) was a red solid powder with molecular weight of 1046.51 (MS: m/z calculated for C_52_H_60_ClN_5_O_16_: 1046.52, found: 1046.50), chemical purity of 99.1% (by HPLC), structure of C_52_H_60_ClN_5_O_16_ (by both of H^1^-NMR and C^13^-NMR) (Figure 2) and melting point of 180°C (decomposition), which is stable at -5°C to 0°C for 36-54 months, ambient temperature for 24 months, solvable in water, partially solvable in methanol and ethanol. PDOX used in this study was stored in the dark, dry area at 4°C.

### PC model construction and histopathological characteristics

**Figure 3:**
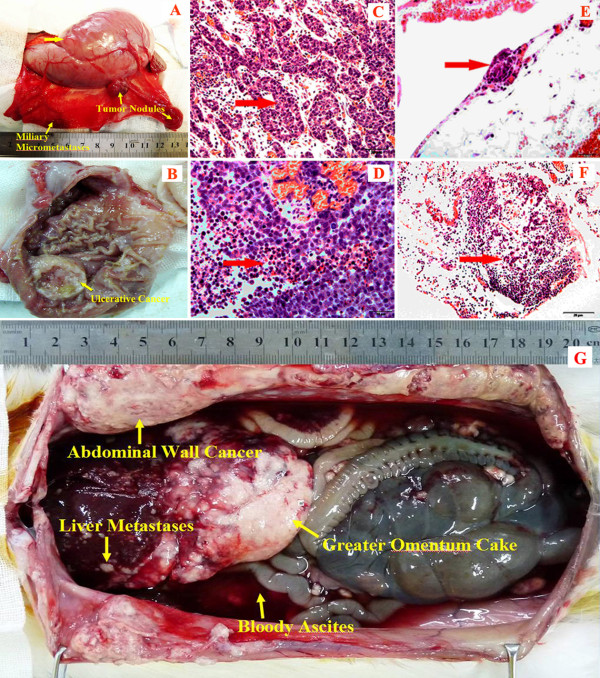
**The characteristics of rabbit model of gastric PC and the HE stained micrographs. (A)** The picture of early rabbit model of gastric PC where conspicuous tumor nodules scattered on the peritoneum and the greater curvature of the stomach; **(B)** the pictures of ulcerative GC; **(C&D)** HE stained picture under microscope showing the invasive growth of tumor cell nests infiltrating the gastric wall (panel **C**, 400×) and necrotic tumor cells in the area of insufficient blood supply (panel **D**, 400×); **(E)** tumor nodules on the surface of the omentum (200×); **(F)** intraperitoneal lymph node metastases (200×); **(G)** post mortem pathological examinations of a rabbit.

Rabbit model of gastric PC was established in all animals (100%, 40/40). On d 8 after tumor cells inoculation, small, hard and transparent tumor nodules developed on the greater omentum, and typical ulcerative cancer about 0.5-1.0 cm in diameter formed on the antrum of the stomach. No ascites was observed. No obvious PC was found in other regions. There were no differences in the PC severity among all the rabbits. This could be equivalent to clinical stage I PC by Gilly criteria [22] (Figure 3A).All investigated tumor specimens showed extensive invasive growth and tissue destruction. The tumors, on the greater curvature of the gastric antrum, penetrated the mucosal layer to form ulcers. Histopathological study showed tumor nests penetrating the entire stomach wall, with typical invasion into the muscle layer and the gastric glands (Figure 3B). The tumor cells are round, oval or atypical morphology with many pathological mitotic figures. There were also conspicuous infiltration of lymphocytes, plasma cells and other inflammatory cells (Figure 3C). Apoptotic and necrotic tumor cells were observed in the central region of the tumor nodules (Figure 3D). Typical PC presented as tumor nodules on the surface of the omentum (Figure 3E) and intraperitoneal lymph node metastases were also observed (Figure 3F).Typical ulcerative cancer with PC was observed in post mortem pathological examinations of rabbits in the Control. The stomach wall was totally invaded by the tumor to create cancer ulcer encased by confluent nodules on the greater omentum, forming a big tumor block. The abdominal wall and diaphragm were totally invaded by the tumor. Many tumor nodules formed on the intestinal wall, the mesentery and the retroperitoneum. Bloody ascites could be more than 100 mL. All the features are similar to the clinicopathologic characteristics of gastric PC in patients (Figure 3G).

### Survival

**Figure 4:**
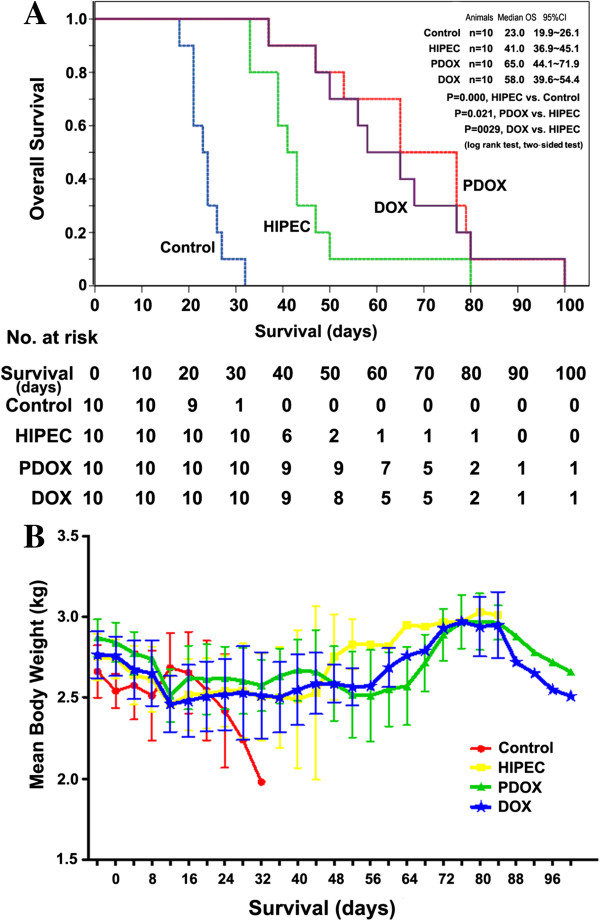
**In vivo effects of PDOX on rabbit PC model. (A)** Kaplan-Meier survival curves for the Control, HIPEC, PDOX and DOX; **(B)** Body weight changes in those groups.

Animal was observed to record OS. Two long surviving rabbits (one in the PDOX and another in the DOX, living more than 100 d) were euthanized on the 100th day by an overdose injection of 2% pentobarbital sodium. The median (95% confidence interval, CI) OS was 23.0 d (19.9 - 26.1 d) in the Control, 41.0 d (36.9 - 45.1 d) in the HIPEC, 65.0 d (44.1 - 71.9 d) in the PDOX and 58.0 d (39.6-54.4 d) in the DOX. Compared with the Control, the OS was extended by at least 70% in the HIPEC (*p* < 0.001, log rank test). Compared with the HIPEC, the OS was further extended by 40% in the DOX (*p =* 0.029, log rank test) and by 58% in the PDOX (*p =* 0.021, log rank test) (Figure 4A).

### Tumor burden at the survival endpoint

At the study endpoint, the tumor burden in the Control was heaviest, with tumor weight of (137.51 ± 16.09) g, tumor-bearing ratio (tumor weight to body weight) of (7.88 ± 0.85)%, bloody ascites of (65.50 ± 33.45) mL, and ePCI score of (9.50 ± 2.17). Compared with the Control, the HIPEC had significantly reduced tumor weight (76.50 ± 11.41) g (*p =* 0.007) and tumor-bearing ratio (3.94 ± 0.54)% (*p =* 0.001), but not ePCI score (9.50 ± 2.17)% (*p =* 0.420) and bloody ascites [(29.16 ± 15.30) mL, *p =* 0.085]. Compared with the HIPEC, PC severity was further decreased in both the PDOX and the DOX, with ePCI score (6.40 ± 2.07, *p =* 0.020 in both PDOX and DOX vs. HIPEC) and bloody ascites (0.00 mL, *p* < 0.001 in PDOX and DOX vs. HIPEC); while there were no significant differences in tumor weight [(65.30 ± 14.55) g, *p =* 0.552, PDOX vs. HIPEC; (48.74 ± 16.31) g, *p = *0.180, DOX vs. HIPEC)] and tumor-bearing ratio[(2.91 ± 0.69)%, *p =* 0.552, PDOX vs. HIPEC; (2.13 ± 0.78)%, *p =* 0.180, DOX vs. HIPEC]. There were no significant differences in tumor burden between the PDOX and the DOX (Table 1).

**Table 1:** The ePCI score at survival endpoint in 4 groups of rabbits [range (median)]

	**Control (n = 10)**	**HIPEC (n = 10)**	**PDOX (n = 10)**	**DOX (n = 10)**
The ePCI score in different regions				
Region I	0 ~ 3 (3)	0 ~ 3 (2)	0 ~ 3 (0)	0 ~ 3 (0)
Region II	3 ~ 3 (3)	3 ~ 3 (3)	3 ~ 3 (3)	3 ~ 3 (3)
Region III	1 ~ 3 (3)	0 ~ 3 (3)	0 ~ 3 (1)	0 ~ 3 (1)
Region IV	0 ~ 3 (3)	0 ~ 3 (1)	0 ~ 3 (0)	0 ~ 3 (0)
Ascites (mL)*	12.0 ~ 113.4 (56.0)	0.0 ~ 94.0 (18.1)	0.0 ~ 0.0 (0.0)	0.0 ~ 0.0 (0.0)
The ePCI score^§^	7 ~ 13 (11)	5 ~ 12 (9)	3 ~ 9 (6)	3 ~ 9 (6)

*Ascites: *p* = 0.323, HIPEC vs. Control; *p* < 0.001, PDOX vs. HIPEC; *p* < 0.001, DOX vs. HIPEC.

^§^The ePCI score: *p* = 0.073, HIPEC vs. Control; *p* = 0.020, PDOX vs. HIPEC; *p* = 0.020, DOX vs. HIPEC; *p* = 0.474, PDOX vs. DOX.

### Body weight changes

The body weight of each animal was recorded every 4 d. No significant differences were found in initial body weight of 4 groups before the treatment. Perioperative body weight decreased in all groups because of the overnight fasting. In the Control, the body weight recovered once food intake was resumed but again decreased progressively till the study endpoint. In the 3 treatment groups, postoperative body weight decreased considerably after model construction and to the lowest 4 d after CRS + HIPEC, and then stayed at a low level during the post-operative phase until chemotherapy had been completed. The body weight began to increase at d 44 in the HIPEC and d 56 in both the PDOX and the DOX, and gradually increased to a higher level, which was related to both tumor growth of the living tumor-bearing rabbits and death of the failure rabbits with lesser body weight. Thereafter, body weight decreased progressively again until the study endpoint in both the PDOX and the DOX (Figure 4B).

### Postmortem pathological examinations

**Figure 5:**
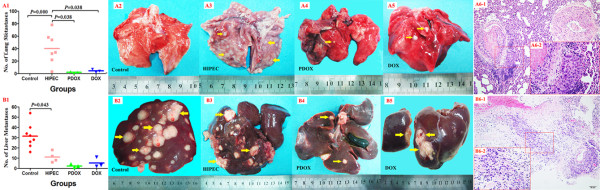
**Pulmonary metastases (panel A) and liver metastases (panel B) in this study. (A1)(B1)** showed exact number of lung and liver metastases in each rabbit of the 4 groups, respectively; (**A2** to **A5**) showed representative lung metastases in the Control, the HIPEC, the PDOX and the DOX, respectively; (**B2** to **B5**) showed representative liver metastases in the Control, the HIPEC, the PDOX and the DOX, respectively; **(A6)** showed micrographs of lung metastases in the HIPEC, characterized by cancer invasion, pulmonary tissue suppression, intravascular tumor thrombus formation and conspicuous inflammatory cells infiltration (**A6-1**, HE stain, 200×)(**A6-2**, HE stain, 400×). **(B6)** showed micrographs of liver metastases in the Control, characterized by extensive liver tissue destruction, invading cancer cells, inflammatory cells infiltration, and hemorrhage in liver tissue (**B6-1**, HE stain, 100×)(**B6-2**, HE stain, 200×).

By the time of animal death, detailed information on postmortem pathological examinations was listed in Table 2.In addition to systematic examinations of all anatomic sites for possible cancer metastases, particular attention was paid to lung and liver metastases. As shown in Figure 5, in the Control, extensive liver metastases were observed in 8 animals, but no pulmonary metastases were evident in all animals, because of the very aggressive growth behaviors of the tumor led to short OS in this group of animals (Figure 5 A2, B2). In the HIPEC, as animals lived much longer because of CRS + HIPEC, fewer liver metastases were observed in 4 animals, but considerably much more pulmonary metastases were observed in 7 animals (Figure 5 A3, B3). In the PDOX and DOX, animals developed much fewer metastases in both the livers and the lungs, although the OS was further improved remarkably, because the animals received not only CRS + HIPEC to control local regional metastases but also systemic chemotherapy to control hematogenous metastases and lymphatic metastases (Figure 5 A4, B4, A5, B5).

**Table 2:** Results of post mortem pathological study in 4 groups, expressed as % of rabbits

	**Control (n = 10)**	**HIPEC (n = 10)**	**PDOX (n = 10)**	**DOX (n = 10)**	***p***_**1**_	***p***_**2**_	***p***_**3**_	***p***_**4**_
Ulcerative GC	100.0	100.0	100.0	100.0	NS	NS	NS	NS
Incision metastases	100.0	30.0	30.0	20.0	0.001	NS	NS	NS
Pulmonary metastases	0.0	70.0	30.0	30.0	0.001	NS	NS	NS
Chest wall metastases	0.0	20.0	10.0	0.0	NS	NS	NS	NS
Pleural effusion	10.0	70.0	30.0	30.0	0.008	NS	NS	NS
Virchow lymph nodes metastases	30.0	70.0	60.0	40.0	NS	NS	NS	NS
Pericardium metastases	0.0	20.0	0.0	0.0	NS	NS	NS	NS
Cancerous diaphragm	100.0	70.0	20.0	20.0	NS	0.028	0.028	NS
Greater omentum cake	100.0	80.0	40.0	50.0	NS	NS	NS	NS
Liver metastases	80.0	40.0	30.0	30.0	NS	NS	NS	NS
Spleen metastases	0.0	0.0	20.0	0.0	NS	NS	NS	NS
Retroperitoneum metastases	100.0	60.0	60.0	50.0	0.029	NS	NS	NS
Left renal metastases	0.0	10.0	0.0	0.0	NS	NS	NS	NS
Right renal metastases	0.0	0.0	0.0	0.0	NS	NS	NS	NS
Kidney capsule invasion	100.0	50.0	0.0	20.0	0.012	0.012	NS	NS
Intestine wall seeding	100.0	80.0	20.0	10.0	NS	0.009	0.002	NS
Mesentery seeding	100.0	80.0	30.0	10.0	NS	0.028	0.002	NS
Abdominal wall cancer	100.0	60.0	30.0	0.0	0.029	NS	0.004	NS
Pelvic seeding	100.0	50.0	30.0	10.0	0.170	NS	NS	NS
Bladder rupture	0.0	0.0	20.0	10.0	NS	NS	NS	NS
Urine retention	50.0	50.0	30.0	10.0	NS	NS	NS	NS

By 2-sided chi-square (*χ*^2^) test. *p*_1_, HIPEC vs. Control; *p*_2_, PDOX vs. HIPEC; *p*_3_, DOX vs. HIPEC; *p*_4_, PDOX vs. DOX.

NS: no significance.

Compared with the Control, PC severity was significantly reduced in incision metastases, pulmonary metastases, pleural effusion, retroperitoneal metastases, kidney capsule invasion, abdominal wall cancer and pelvic seeding in the HIPEC. Compared with the HIPEC, PC severity was significantly reduced in the cancerous diaphragm, kidney capsule invasion, intestine wall seeding and mesentery seeding in the PDOX, and in the cancerous diaphragm, intestine wall seeding, mesentery seeding and abdominal wall cancer in the DOX. There were no statistical differences between the PDOX and DOX in systemic metastases.

### Peripheral blood profile and major biochemistry changes

There were no differences in peripheral blood cell counts on d 2, d 6 and d 14 among the 4 groups, but the white blood cells and platelets were significantly decreased on d 36 after systemic chemotherapy in the DOX (*p* < 0.05). There were no differences in the hepatic and renal functions throughout the experiment among the 4 groups. In terms of cardiac functions parameters, there were no differences in creatine kinase and creatine kinase mb isoenzyme on d 2, d 6 and d 14 among the 4 groups, but creatine kinase mb isoenzyme was significantly increased on d 36 after systemic chemotherapy in the DOX (*p* < 0.05). Of particular attention, 2 animals in the DOX with short OS of 47 d and 53 d had exceptionally higher serum creatine kinase mb isoenzyme levels, 467.5 U/L and 656.4 U/L, respectively (*p* < 0.05) (Table 3).

**Table 3:** Blood routine tests and biochemical test results in 4 groups of rabbits [range (median)]

**Group**	**D2**			**D6**				**D14**		D36*		
	**n**	**Value**	***p***	**n**	**Value**	***p***	**n**	**Value**	***p***	**n**	**Value**	***p***
Red blood cells (T/L)												
Control	10	5.03-6.93 (6.26)	0.849	10	5.08-7.76 (6.07)	0.494	10	4.29-6.32 (5.54)	0.382	0	-	0.203
HIPEC	10	5.13-7.58 (6.48)		10	4.66-7.15 (5.65)		10	3.54-7.30 (5.68)		7	4.28-7.36 (5.28)	
PDOX	10	4.17-7.16 (5.94)		10	4.97-6.49 (5.61)		10	4.38-5.82 (5.05)		10	4.46-6.03 (5.47)	
DOX	10	5.03-6.84 (6.09)		10	5.03-6.70 (5.88)		10	3.65-6.36 (5.08)		9	4.12-6.40 (4.94)	
White blood cells (G/L)												
Control	10	5.3-9.6 (8.6)	0.785	10	4.5-14.1 (9.9)	0.446	10	6.0-10.3 (7.8)	0.601	0	-	0.013
HIPEC	10	4.4-12.3 (7.4)		10	4.5-12.7 (9.3)		10	3.2-12.1 (10.7)		7	6.9-13.7 (7.5)	
PDOX	10	5.9-13.2 (8.2)		10	6.1-12.5 (8.8)		10	4.6-14.4 (9.0)		10	4.9-11.1 (7.7)	
DOX	10	3.6-10.5 (7.8)		10	4.7-12.2 (7.3)		10	4.6-12.5 (10.2)		9	3.9-6.4 (5.3)	
Platelets (G/L)												
Control	10	387-579 (480)	0.494	10	317-836 (555)	0.362	10	451-917 (657)	0.601	0	-	0.009
HIPEC	10	229-634 (373)		10	363-758 (408)		10	390-709 (489)		7	262-598 (451)	
PDOX	10	382-592 (456)		10	395-856 (493)		10	328-1002 (500)		10	201-532 (363)	
DOX	10	330-647 (476)		10	363-878 (464)		10	390-939 (654)		9	115-319 (244)	
Alanine transaminase (U/L)												
Control	10	31-52 (38)	0.463	10	34-54 (41)	0.525	10	21-54 (37)	0.245	0	-	0.838
HIPEC	10	24-47 (40)		10	21-62 (35)		10	31-66 (42)		7	30-59 (48)	
PDOX	10	28-52 (40)		10	24-59 (37)		10	32-55 (45)		9	25-79 (40)	
DOX	10	29-59 (35)		10	30-59 (39)		10	34-61 (48)		8	26-76 (42)	
Aspartate transaminase (U/L)												
Control	10	19-54 (35)	0.895	10	30-52 (36)	0.525	10	37-58 (43)	0.309	0	-	0.473
HIPEC	10	23-59 (38)		10	11-55 (35)		10	27-78 (34)		7	25-50 (36)	
PDOX	10	22-47 (37)		10	17-49 (39)		10	27-69 (36)		9	20-41 (30)	
DOX	10	24-47 (37)		10	24-49 (30)		10	23-86 (36)		8	31-60 (39)	
Blood urea nitrogen (mmol/L)												
Control	10	4.40-5.80 (4.80)	0.206	10	4.51-10.44 (4.81)	0.525	10	3.29-9.82 (6.32)	0.185	0	-	0.473
HIPEC	10	3.70-8.14 (5.10)		10	3.57-11.04 (5.35)		10	4.32-9.29 (7.50)		7	5.25-8.67 (6.38)	
PDOX	10	3.58-7.66 (5.82)		10	3.32-11.58 (6.68)		10	6.52-13.58 (7.63)		9	4.71-8.05 (5.35)	
DOX	10	1.76-7.90 (5.01)		10	3.32-10.00 (5.59)		10	1.76-10.53 (7.80)		8	4.50-9.26 (6.27)	
Creatinine (μmol/L)												
Control	10	45.05-64.50 (60.50)	0.736	10	22.10-73.20 (55.00)	0.736	10	41.40-71.40 (50.25)	0.083	0	-	0.838
HIPEC	10	43.30-80.65 (57.35)		10	22.20-81.55 (46.97)		10	31.10-83.37 (52.96)		7	59.87-76.84 (66.51)	
PDOX	10	42.80-75.80 (61.87)		10	22.13-86.25 (69.65)		10	45.30-75.80 (66.25)		9	44.38-79.56 (62.47)	
DOX	10	22.30-69.30 (55.81)		10	27.28-75.90 (63.45)		10	42.38-73.76 (69.37)		8	47.72-78.64 (59.50)	
Creatine kinase (U/L)												
Control	10	74.3-205.6 (131.6)	0.864	10	81.3-247.8 (147.9)	0.878	10	71.4-182.1 (138.9)	0.968	0	-	0.473
HIPEC	10	78.8-259.2 (137.8)		10	66.8-248.9 (145.3)		10	63.4-225.2 (141.8)		7	98.3-265.1 (153.0)	
PDOX	10	87.9-194.2 (139.4)		10	69.9-256.1 (144.4)		10	70.0-240.8 (130.6)		9	109.2-251.5 (195.2)	
DOX	10	98.3-256.1 (152.4)		10	56.4-250.2 (135.8)		10	80.5-261.2 (133.8)		8	101.1-407.6 (228.1)	
Creatine kinase mb isoenzyme (U/L)												
Control	10	73.0-162.5 (129.8)	0.717	10	62.5-195.6 (127.2)	0.878	10	57.0-199.8 (119.8)	0.509	0	**-**	0.040
HIPEC	10	74.2-228.2 (137.3)		10	42.9-219.8 (129.7)		10	52.8-237.7 (146.5)		7	50.2-188.1 (126.1)	
PDOX	10	59.2-228.1 (133.9)		10	42.5-197.7 (133.2)		10	74.9-241.5 (158.2)		9	58.9-209.8 (143.7)	
DOX	10	94.3-207.7 (122.1)		10	53.9-202.3 (128.2)		10	94.3-238.6 (148.4)		8	106.2-656.5 (259.3)	

*Part of the data is not available because of animal death at the set time.

By 2-sided nonparametric test.

White blood cells on D 36, comparison between groups: *p =* 0.001, DOX vs. HIPEC; *p =* 1.000, PDOX vs. HIPEC; *p =* 0.005, PDOX vs. DOX; Platelets on D 36, comparison between groups: *p =* 0.041, DOX vs. HIPEC; *p =* 0.637, PDOX vs. HIPEC; *p =* 0.070, PDOX vs. DOX; Creatine kinase mb isoenzyme on D 36, comparison between groups: *p =* 0.012, DOX vs. HIPEC; *p =* 0.593, PDOX vs. HIPEC; *p =* 0.131, PDOX vs. DOX.

Hematological toxicities with marked reduction in white blood cells (*p* < 0.05) and platelets (*p* < 0.05), as well as cardiac toxicities with significant increases in creatine kinase mb isoenzyme (*p* < 0.05) were observed on d 36 after systemic chemotherapy in the DOX.

### Detailed studies on myocardium toxicities

**Figure 6:**
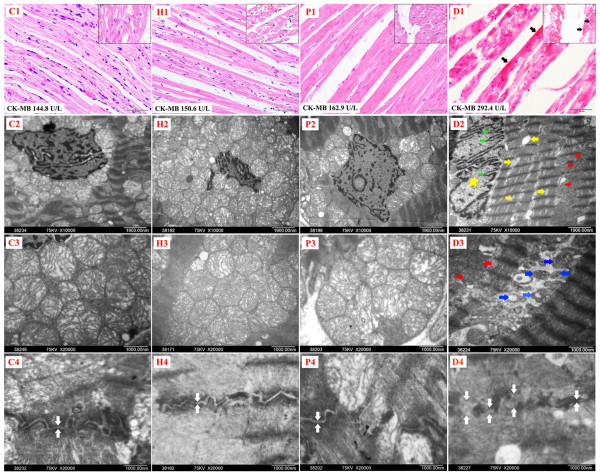
**Light micrographs and electron transmission micrographs of rabbit myocardium.** Top panel (HE stain, 400×), routine histopathological features of rabbit myocardium at longitudinal section (inserts on the upper right corner, sagittal section), where **C1**, **H1** and **P1** demonstrated clean and clear normal myocardial fibers in orderly arrays, but **D1** showed coagulation necrosis of the myocardium with deep red stained cytoplasm and the karyopyknosis of the myocardium. Middle upper panel (10,000×), electron transmission micrographs of the myocardium focusing on the features of nuclei and peri-nuclear mitochondria, where **C2**, **H2** showed abundant mitochondria packed around normal nucleus, **P2** showed rich mitochondria scattered around normal nucleus, but **D2** showed nearly absent mitochondria around the morphologically senile and degenerative nucleus (yellow star showing enlarged nucleus due to swelling and the eccentric aggregation of heterochromatin, and green arrows showing conspicuous inward contraction of the nuclear membrane ). In addition, partial myocardium-lytic necrosis (yellow arrow) and mitochondria-pyknosis away from the nucleus (red arrow) were also evident in DOX treated animals. Middle lower panel (20,000×), electron transmission micrographs of the myocardium specifically focusing on the detailed morphological changes of mitochondria, where **C3** and **H3** showed normal mitochondria morphology, **P3** showed normal mitochondria morphology but with slightly dilated cristae in some mitochondria, but **D3** showed conspicuous mitochondria-pyknosis with significantly smaller size and highly packed cristae (red arrows) and completely disintegrated mitochondria reducing to high electron-density bodies (blue arrows). Bottom panel (20,000×), electron transmission micrographs of the myocardium focusing on the detailed morphological changes of intercalated discs, where **C4**, **H4** and **P4** showed complete, clear and continuous lines of normal intercalated discs (white arrows), but **D4** showed obscure, loosing and discontinuous intercalated discs (white arrows).

Histopathological studies under both light microscope and transmission electron microscope found no myocardium toxicities in the Control, the HIPEC and the PDOX, but significant histopathological changes of myocardium were found in all animals (100%, 10/10) in the DOX, including coagulation necrosis of the myocardium with deep red stained cytoplasm, and the karyopyknosis (Figure 6 C1, H1, P1, D1 black arrows). Electron transmission micrographs showed that normal myocardium with abundant mitochondria packed around normal nucleus in the Control and HIPEC, rich normal but with slightly dilated cristae mitochondria scattered around normal nucleus in the PDOX, nearly absent mitochondria around the morphologically senile and degenerative nucleus in the DOX. In addition, partial myocardium-lytic necrosis (yellow arrow) and mitochondria-pyknosis away from the nucleus (red arrow) were also evident in the DOX. Complete, clear and continuous lines of normal intercalated discs in the Control, the HIPEC and the PDOX, but obscure, loosing and discontinuous intercalated discs were found in the DOX (white arrows).

## Discussion

There is no standard treatment for gastric PC. CRS plus HIPEC represent a multidisciplinary approach to this problem. It was first reported in 1988 by Fujimoto et al [23] on 15 patients with PC secondary to advanced GC, with a mean survival of 7.2 ± 4.6 months with acceptable morbidity. This new treatment modality gradually gains acceptance in many countries. The reported studies use different PCI scoring system to evaluate the extent of PC and different HIPEC approaches, but they produce similar results that CRS + HIPEC provides treatment benefit. In order to more objectively evaluate such treatment, it is necessary to study this treatment modality under experimental conditions, in which most of the confounding factors could be well controlled.

In our previous study [24,25], we have established a stable rabbit model of gastric PC by injecting VX2 cancer cells into the sub-mucosal layer of the stomach. The non-immuocompromised larger animal model is characterized by typical ulcerative GC with progressive PC, making it more suitable for surgical interventional studies to evaluate CRS and HIPEC against gastric PC. This rabbit model of gastric PC has provided us with suitable platform to evaluate different therapeutic approaches against PC. On this first large animal model of gastric PC, we proved that CRS + HIPEC with docetaxel and carboplatin could significantly prolong the OS by at least 60% (40 d vs. 23 d) with acceptable safety. The addition of molecular targeted therapy with PDOX could provide much better survival benefits with satisfactory drug safety.

This study provided new approach of CRS + HIPEC combined with molecular targeted therapeutic regimen PDOX to gastric PC. Compared with the Control, the OS was extended by at least 70% (18 d) in the HIPEC (*p* < 0.001). Compared with the HIPEC, the OS was extended by at least 40% (17 d) with DOX therapy (*p =* 0.029) and by 58% (24 d) with PDOX treatment (*p =* 0.021).

In currently available chemotherapeutic regimens, anthracyclines are important drugs, as is well demonstrated in the MAGIC trial [26-28]. Anthracyclines cause cell damage by intercalating into DNA, leading to chromatin unfolding and aggregation, which ultimately results in apoptosis [29]. However, like many cytotoxic agents, anthracyclines can cause serious organ damages. With DOX, toxicity to the heart and bone marrow are usually dose-limiting, with the maximum tolerated dose (MTD) far below the minimum curative dose (MCD). Therefore, strategies to shield the heart and bone marrow by excluding DOX from them have long been a top priority. Another strategy has been to target the drug to the tumor by attaching it to some tumor-binding moiety, e.g. a tumor-specific monoclonal antibody (MAb) such as trastuzumab used in chemotherapy for HER2-positive GC [30]. Drawbacks to the use of MAbs have been that (1) the limited diffusion of MAbs due to their large macromolecules into solid tumors could compromise their potency; (2) heterogeneous antigen expression in large tumors will evidently lead a portion of tumor cells in the solid tumor unresponsive to MAbs; (3) the tumor Ag that binds the MAb is never completely tumor-specific, so that some of the drug goes where it does harm; (4) foreign MAbs are often immunogenic; and (5) MAbs therapy is very expensive [31-33].

In the present study, normal organs are protected by masking the cytotoxic drug DOX with a simple dipeptide that renders it nontoxic. At the tumor the mask is removed by CTSB, a ubiquitous proteolytic enzyme that is so destructive to tissue that normally it occurs only within cells, encased in lysosomes [34]. Only tumor cells secrete CTSB externally, confined to their plasma membranes, for the purpose of penetrating basement membrane and extracellular barriers as they spread [35]. The prodrug PDOX is rapidly cleaved by CTSB at the Lys-PABC bond [16]. The resulting PABC-DOX decomposes at once to para-aminobenzyl alcohol, CO_2_ and free DOX [16,21]. The PABC self-immolating linker is necessary because the CTSB’s active site cannot accommodate the bulky DOX molecule, but the smaller PABC fits into the active site [16]. Free DOX released right on the tumor cells penetrates them readily, killing them. To be sure, a certain portion of the free drug may drift away from the tumor, but the concentration ratio tumor/heart-bone marrow should be much higher than if the DOX is given as the free drug, when the expected ratio is about 1. In this way, even without a positive targeting agent like a MAb it is possible to raise the MTD without significantly raising the MCD. The goal is to raise the MTD above the MCD, where cures become possible, but even short of that, a rise in the MTD/MCD ratio will enhance the effect.

In the previous study [16], human plasma stabilities of PDOX and releasing rates of free DOX were measured. The results indicated that PDOX was not hydrolyzed (no observable changes) over 6 - 7 h in human plasma without CTSB, but hydrolyzed in 16 min in CTSB rich conditions, and the appearance of DOX correlated precisely with the disappearance of PDOX at 495 nm, suggesting that PDOX could be stable in blood circulation and effectively released at the CTSB-rich tumor site. The evidence implies that PDOX could stay relatively stable in human blood circulation for a reasonable time, but the rabbit plasma stability of PDOX was not measured in this study.

DOX is one of the most efficacious anticancer drugs with limited usage due to its dose-dependent toxicities to the heart, kidney, liver and bone marrow. Over the past years, a number of drug delivery strategies have been developed in an attempt to improve efficacy and reduce the toxicity profile of DOX. The most successful drug delivery strategy reported to date has been liposomal DOX, which favors accumulation of the drug at tumor sites because liposomes easily exit the bloodstream at sites of leaky vasculature but do not readily exit the circulation in healthy tissues, such as the heart, with its “tight” endothelial capillary junctions [36]. Such theoretical advantages have been confirmed by phase III clinical studies on metastatic breast cancer, which demonstrated that liposomal DOX could achieve similar or slightly better clinical efficacy but with significantly reduced cardiac and hematological toxicities, in comparison with conventional DOX [37,38]. Compared with liposomal DOX, PDOX is a structurally different molecule designed for different antitumor mechanisms. Since we did not perform direct comparisons of efficacies and side effects between these two agents, it is likely that PDOX would not have an advantage in cardiac toxicity if it were compared to liposomal DOX.

In this study, animals receiving DOX treatment showed significant hematological toxicities with marked reduction of white blood cells and platelets, as well as cardiac toxicities with significant increases in creatine kinase mb isoenzyme, which is sensitive marker of cardiac toxicities, evident myocardium coagulation necrosis under histopathological studies. No hepatic and renal toxicities were observed in the DOX. In contrast, these toxic effects were not evident in animals receiving PDOX treatment.

In previous studies, TEM revealed DOX-induced following myocardial damages in rabbits: sarcoplasmic vacuolization, mitochondrial disruption, and myofibrillar lysis, which became more serious along with the increase of DOX dose [39], while demonstrated marked mitochondrial damage and vacuolization in DOX-treated mouse hearts [40]. Disruption or loss of myofibrils and vacuolization of the cytoplasm, mitochondria changes, patchy necrosis, and inflammatory cells were observed in DOX-treated rats [41]. Heavily contracted fibrils and condensed mitochondria were seen in TEM of heart cells in DOX-treated dogs [42]. In this study, DOX induced mitochondria-centered cardiac injuries involving significant nuclear degenerative changes, mitochondria deletion in the peri-nucleus region, mitochondria-pyknosis, and discontinuation of intercalated discs under electron microscopy. These results were similar to the cardiac toxicity profiles in the above literatures. However, such toxic effects were not evident in the PDOX, even though the dosage of PDOX was 5.55-fold that of DOX. In addition, the hematologic, hepatic and renal toxicities were also less evident in PDOX than in DOX. Therefore, PDOX demonstrated reduced overall toxicity in comparison with DOX.

In conclusion, this study successfully synthesized PDOX with high chemical purity and good water solvability. PDOX is a newly synthesized molecular targeting prodrug of DOX, with defined chemical-physical properties. Compared with DOX, PDOX has similar efficacy but reduced hematological and cardiac toxicities in treating rabbit model of gastric PC.

## Methods

### Synthesis and identification of PDOX

All reactions were carried out in dried flasks under an atmosphere of dry nitrogen unless otherwise specified. The reactions monitored either on HPLC or TLC for completion. All reagents and solvents used were used as received without further purification unless otherwise stated. Chemical purity of the intermediates and final compound was determined on Agilent 1100 series instrument. LC-MS was performed on Agilent 1100 instrument with Zorbax column (4.6 × 150 mm) eluted with 0.1% trifluoroacetic acid water/0.1% trifluoroacetic acid acetonitrile at 1 mL/min flow rate. Column chromatography was performed on silica gel 60 (40-63 μm). Nuclear magnetic resonance (NMR) spectra were recorded on a Varian 300 instrument operating at 300 MHz (^1^H) or Bruker AC 500 instrument operating at 500 MHz (^1^H), 125 MHz (^13^C). Chemical shifts δ were reported in parts per million (ppm) from tetramethylsilane (TMS) as internal standards (0.00 ppm). Abbreviations for signal coupling are as follows: s = singlet, d = doublet, t = triplet, q = quartet, dd = double doublet, m = multiplet. High resolution mass spectrum (ES, positive) was determined on a Thermo LTQ FT Ultra Mass Spectrometer.

PDOX was synthesized according to the previously reported chemical process [16-18], with the following 7 major steps.

#### Step-1: Fmoc-Lys(MMT) 1

In a 2 L r.b. flask, Fmoc-L-Lysine hydrochloride (47.6 g, 112.8 mmol) was suspended in anhydrous dichloromethane (500 mL). Under nitrogen, at room temperature the trimethylchlorosilane (30 mL, 2.1 eq) was added, followed by diisopropylethylamine (20.6 mL, 1.05 eq). The reaction mixture was then heated to reflux for 1 h, and the clear solution formed during this time. The reaction mixture was then cooled to 0°C, and the diisopropylethylamine (62 mL, 3.1 eq) was added in, followed by p-anisyl-diphenylmethyl chloride (36.6 g, 1.05 eq). The reaction mixture was stirred at room temperature for 16 h. The solvent was evaporated and the residue was re-dissolved in ethyl acetate (600 mL). The solution was washed twice by pH 5 buffer solution (biphosphate, 300 mL × 2), and dried over sodium sulfate. After filtration and evaporation, a foam solid obtained (69.4 g 96%). H^1^-NMR (CDCl_3_, ppm) 1.26 (m, 2H), 1.68 (m, 4H), 2.45 (m, 2H), 3.71 (s, 3H), 4.05-4.40 (m, 4H), 6.81 (d, 2H), 7.15-7.77 (m, 20H).

#### Step-2: Lys(MMT) 2

In a 1 L flask, under nitrogen, Fmoc-Lys(MMT) 1 (26.3 g, 41 mmol) was dissolved in a mixture of 1:1 dichloromethane acetonitrile (400 mL). The diethylamine (400 mL) was added at room temperature and the result solution was stirred for 1.5 h. The solvent was evaporated to dryness, and the residue was treated with acetonitrile (200 mL) and the ether 400 mL. the solid was collected by filtration. The solid crude product was stirred with a mixture solvent of dichloromethane methanol (100 mL: 20 mL). The solid side product was filtrated off. After evaporation the solid product was dried by high vacuum for 10 h and obtained 16 g (94%). H^1^-NMR (DMSO-d6, ppm) 1.34-1.72 (m, 6H), 2.05 (m, 2H), 3.38 (m, 1H), 3.68 (s, 3H), 3.71 (d, 2H), 7.03-7.40 (m, 12H).

#### Step-3: Ac-Phe-Lys(MMT) 3

In a 1 L flask under nitrogen, the Lys(MMT) 2 (20.5 g, 48.9 mmol) was dissolved in dimethoxyethane (350 mL), and the solution of lithium hydroxide (2.05 g, 1 eq) in water (125 mL) was added in. A solution of Ac-Phe-OSu (13.9 g, 1 eq) in dimetoxyethane (350 mL) was added during 15 min, and the reaction mixture was stirred at room temperature for 16 h. The solvent was evaporated as much as possible, and the residue was taken by ethyl acetate (350 mL). This solution was washed by pH 4 buffer solutions (250 mL × 2) and brine (100 mL), and dried over sodium sulfate. After evaporation, the pale-yellow solid was dried by high vacuum for at least 5 h, and obtained 26.5 g (90%). H^1^-NMR (CDCl_3_/CD_3_OD), ppm) 1.22 (m, 2H), 1.58 (m, 3H), 1.71 (m, 1H), 1.82 (s, 3H), 2.49 (m, 2H), 3.00 (m, 2H), 3.75 (s, 3H), 4.26 (t, 1H), 4.63 (t, 1H), 6.82 (d, 2H), 7.10-7.43 (m, 17H).

#### Step-4: Ac-Phe-Lys(MMT)-PABOH 4

In a 1 L flask under nitrogen, Ac-Phe-Lys(MMT) 3 (26.5 g, 43.65 mmol) and di-t-butyl-pyrocarbonate (14.3 g, 1.5 eq) were dissolved in dichloromethane (600 mL), and at room temperature, the pyridine (3.7 mL, 1.05 eq) was added in. The resulting mixture was stirred for 15 min, and then, p-aminobenzyl alcohol (8 g, 1.5 eq) was added in. The reaction was stirred for 16 h, and the solvent was evaporated to dryness. The residue was treated with ether (200 mL), and the solid product was collected by filtration, and washed by fresh ether (50 mL × 2). After drying by high vacuum for 10 h, the off white solid obtained 26.2 g (84%). H^1^-NMR (CDCl_3_/CD_3_OD), ppm) 1.31 (m, 1H), 1.50 (m, 1H), 1.71-2.01 (m, 7H), 2.18 (m, 2H), 3.00 (m, 2H), 3.74 (s, 3H), 4.40 (t, 1H), 4.61 (s, 2H), 4.68 (m, 1H), 6.67 (d, 1H), 6.77 (d, 1H), 7.00-7.55 (m, 21H), 8.92 (br, 1H).

#### Step-5: Ac-Phe-Lys(MMT)-PABC-PNP 5

In a 1 L flask under nitrogen, Ac-Phe-Lys(MMT)-PABOH 4 (25.5 g, 35.7 mmol) and bis-p-nitrophenylcarbnate (32.5 g, 3 eq) was dissolved in anhydrous dichloromethane (600 mL). The fresh powdered molecular sieve 4 (50 g) was added followed by addition of diisopropylethylamine (18.5 mL, 3 eq). The reaction mixture was stirred at room temperature for 20 h, and filtrated through a pad of celite. After evaporation of the solvent, the crude compound was loaded to a silica gel column, and the desired product was eluted with hexane-ethyl acetate (50% to 100%). The pure product was obtained after evaporation of solvents, gives 10.5 g (34%). H^1^-NMR (DMF-d7, ppm) 1.43 (m, 2H), 1.58 (m, 2H), 1.72 (m, 1H), 1.87 (m, 4H), 2.09 (m, 2H), 2.4 (br, 1H), 3.05 (m, 1H), 3,78 (s, 3H), 4.52 (m, 1H), 4.72 (m, 1H), 5.36 (s, 2H), 6.90 (d, 2H), 6.77 (d, 1H), 7.29 (m, 16H), 7.41 (d, 2H), 7.50 (d, 4H), 7.68 (d, 2H), 7.81 (d, 2H), 8.13 (s, 1H), 8.19 (d, 1H), 8.41 (d, 2H), 10.11 (s, 1H).

#### Step-6: Ac-Phe-Lys(MMT)-PABC-DOX 6

In a 1 L flask under nitrogen, Ac-Phe-Lys(MMT)-PABC-PNP 5 (11 g, 12.5 mmol) and DOX hydrochloride (7.6 g, 1.05 eq) were dissolved in dimethylformamide (600 mL). The diisopropylethylamine (2.3 mL, 1.05 eq) was added, and the reaction mixture was stirred at room temperature for 48 h. The reaction mixture was diluted with ethyl acetate (4 L), and washed with water (1 L × 4), and dried over sodium sulfate. After evaporation, the crude product was purified by silica gel column and eluted dichloromethane-methanol (5% to 15%). After evaporation and drying, the pure product obtained 9.8 g (61%). H^1^-NMR (DMF-d7, ppm) 1.25 (d, 3H), 1.41 (m, 2H), 1.72 (m, 2H), 1.87 (m, 4H), 2.09 (m, 4H), 2.34 (m, 4H), 3.12 (m, 4H), 3.63 (br, 1H), 3,78 (s, 3H), 3.92 (m, 1H), 4,11 (s, 3H), 4.33 (m, 1H), 4.51 (m, 1H), 4.68 (m, 1H), 4.81 (s, 2H), 4.90 (m, 1H), 5.00 (br, 2H), 5.13 (br, 1H), 5.40 (br, 1H), 5.61 (s, 1H), 6.78 (d, 1H), 6.89 (d, 2H), 7.29 (m, 17H), 7.41 (d, 2H), 7.50 (d, 4H), 7.70 (m, 3H), 8.05 (m, 3H), 9.98 (s, 1H).

#### Step-7: Ac-Phe-Lys-PABC-DOX-HCl 7

In 1 L flask under nitrogen, Ac-Phe-Lys(MMT)-PABC-DOX 6 (9.8 g, 7.6 mmol) was suspended in a mixture of anisole (83 mL, 100 eq) and dichloromethane (260 mL), then dichloroacetic acid (6.2 mL, 10 eq) was added. The reaction mixture was stirred at room temperature for 1.5 h, and the ethyl acetate (2 L) was added, and keep stirring for 2 h. The solid product was collected by filtration, and the solid was re-dissolved in methanol (400 mL). The solution was loaded to a AG2-X8 ion resin (Cl form, 250 g) exchange column, and eluting with methanol. The colorful fraction, which content product, was collected and evaporated to dry. The solid was treated with dichloromethane (10 mL) and after filtration, the product was dried by high vacuum for constant weight produced 7.1 g (95%).

#### H^1^-NMR (DMSO-d6, ppm

1.12 (d, 3H), 1.47 (m, 4H), 1.75(m, 4H), 1.83 (m, 1H), 2.11 (d, 1H), 2.2 (d, 1H), 2.67-3.02 (m, 4H), 3.74 (m, 1H), 3.86 (s, 3H), 4.13 (m, 2H), 4.38, (m, 1H), 4.51 (m, 1H), 4.50 (br, 2H), 4.75 (d, 1H), 4.89 (br, 2H), 5.21 (br, 1H), 5.48 (s, 1H), 6.89 (d, 1H), 7.11-7.26 (m, 4H), 7.59 (d, 1H), 7.64 (dd, 1H), 7.85-7.90 (m, 3H), 8.16, (d, 1H), 8.32 (d, 1H), 10.11 (s, 1H), 13.16 (s, 1H).

#### C13-NMR (DMSO-d6, ppm)

δ 186.6, 186.5, 171.8, 170.8, 170.1, 169.8, 161.1, 156.4, 155.7, 154.9, 138.9, 138.7, 138.3, 137.8, 136.5, 135.7, 134.8, 134.4, 132.3, 129.6, 128.8, 126.7, 126.6, 120.2, 119.5, 119.6, 110.9, 110.8, 100.7, 75.3, 70.1, 68.4, 76.1, 65.3, 64.1, 56.8, 55.1, 54.5, 53.7, 48.9, 47.5, 39.2, 38.8, 36.8, 32.4, 31.6, 30.2, 26.9, 22.8, 22.4, 17.4.

### Agents and cells

Apart from PDOX described above, other agents were obtained from commercial sources, including DOX hydrochloride for Injection (Pharmacia, Milan, Italy), Docetaxel (Wanle Pharmaceuticals Co., Ltd. Shenzhen, China) and Carboplatin (Qilu Pharmaceuticals Co., Ltd. Shandong, China).

### Ethics statement

The animal study protocol was approved by the Animal Welfare Committee of Wuhan University, complied with the Helsinki Declaration. The animal study was completed in the Animal Biosafety Level 3 Laboratory at the Animal Experimental Center of Wuhan University (Animal Study Certificate ID: No.00022310).

### Animals

Forty male New Zealand white rabbits, body weight between 2.5-3.0 kg, were obtained from Animal Biosafety Level 3 Laboratory at the Animal Experimental Center of Wuhan University (Approval ID: SCXK 2008-0004). The animals were individually housed and allowed free access to standard laboratory food and water as well as 12 h of light and dark cycle per day.

### Tumor strain and tumor cell preparation

Rabbit VX2 carcinoma was used to establish GC with PC in this study. The VX2 tumor is a transplantable rabbit squamous cell carcinoma, characterized by rapid tumor growth and early metastasis, established from a virus-induced papilloma by Rous and coworkers [43]. The tumor was maintained by successive in vivo transplantation into the hind leg of a carrier rabbit used for every passage.

When the VX2 tumor grew to about 2 cm in diameter on the carrier rabbit, the animal was anesthetized by ear vein injection of 2% pentobarbital sodium (30 mg/kg). After skin preparation and disinfection, the tumor was excised from the carrier rabbit and placed in icy cold 0.9% sodium chloride solution. Tumor tissue was minced into approximately 1.0-2.0 mm^3^ fragments and suspended in 2 mL of normal saline, then drawn into a 2 mL injector. Other tumor tissues about 3.0-5.0 mm^3^ were placed into the homogenizer embedded in ice bath, to which 3 mL of icy cold normal saline was added, and the tumor cells suspension was made, with the tumor cells concentration adjusted to 5 × 10^10^ vial cells/L.

### Construction of rabbit model of gastric PC

Rabbit VX2 carcinoma was used to establish gastric PC in this study. All rabbits had overnight fasting before experiment, but water was given ad libitum. The animals were anesthetized by ear vein injection of 2% pentobarbital sodium (30 mg/kg). The abdominal skin was cleaned and disinfected. Tumor cells were injected into the stomach submucosa layer to construct rabbit model of gastric PC as described previously [24,25]. Briefly, a midline incision of 3 cm long was made beginning 2 cm below the xyphoid and the upper abdomen was open. The stomach was exposed, 0.1 mL of tumor cells (5 × 10^10^ vial cells/L) was injected into the submucosal layer of the stomach, through the serosal layer and the muscle layer, the injection site was pressed for 1 min to keep the injected tumor cells in place, and the abdomen was closed with a double layer 3-0 vicryl interrupted suture. After tumor inoculation, Penicillin G at the dose of 100,000 IU/d was intramuscularly injected to each animal for 3 d.

### Randomization and treatment

When animal model construction has been confirmed successful on d 8 after operation, these rabbits were randomly divided into 4 groups according to a computer generated randomize number, 10 animals in each group: the Control (n = 10) was observed for natural course of disease progression without any intervention; the HIPEC (n = 10) receiving CRS plus HIPEC (docetaxel 10.0 mg and carboplatin 50.0 mg in 250 mL normal saline, at 42.5 ± 0.5°C for 30 min); the PDOX (n = 10) and the DOX (n = 10) receiving systemic chemotherapy with PDOX 50.0 mg/kg (10.0 mg/kg every 4 d for 5 cycles) or DOX 5.0 mg/kg (1.0 mg/kg every 4 d for 5 cycles) after CRS + HIPEC, respectively; The CRS + HIPEC was performed on d 8 while the systemic chemotherapy was initiated on d 16 after model construction.

### CRS plus HIPEC

For the three treatment groups, CRS + HIPEC was performed on 8 d after tumor cells inoculation. Rabbits were given 2% pentobarbital sodium (30 mg/kg) intravenously (i.v) for anesthesia. The abdominal skin was cleaned and disinfected. The abdominal exploration was performed through a midline incision of 8 cm long beginning 1 cm below the xyphoid. Once the abdomen was open, detailed evaluation of the PC was conducted in different regions including the parietal peritoneum, visceral peritoneum, the omentum, stomach, liver, spleen, intestinal wall, bladder and other pelvic organs. An ePCI score system [44] was developed to record the experimental peritoneal carcinomatosis index of the animals in each group, which took into consideration of tumor nodule sizes, distributions and the characteristics of ascites. In this system, the abdominal cavity of rabbit was divided into 4 regions: region I, sub-diaphragm; region II, the liver, spleen, stomach and affiliated ligaments; region III, small intestine, colon, mesenterium and abdominal wall; and region IV, pelvic cavity urogenital system and rectum. The detailed scoring criteria were modified from similar reporting systems on rat and mice PC models and set as the following: score 0, no tumor nodules throughtout the region; score 1, nodule size less than 5 mm in greastest diameter; score 2, nodule size greater than 5 mm and up to 20 mm; score 3, nodule size greater than 20 mm. If bloody ascites occurred, it was set as score 1. The sum of all the scores was the ePCI of the animal (ranging from 0 to 13). Thereafter, maximal CRS was performed including a routine omentectomy, and optimal removal of tumor nodules. Unresectable tumors were cauterized. The gastric tumor itself, however, was not removed but treated by injection of absolute alcohol. HIPEC was performed just before the closure of abdominal cavity after completion of CRS, as this open technique was believed to provide optimal thermal homogeneity and spatial diffusion [25], with 250 mL of heated saline containing 10 mg of docetaxel and 40 mg of carboplatin for each animal. The abdominal cavity was rinsed twice with 250 mL of normal saline preheated to 42.5°C and perfusion tube was placed in pelvic cavity just before HIPEC. The perfusion equipment consisted of a miniature heat exchanger and a roller pump, allowing perfusion with a variable dynamic flow of 6-12 mL/min. An inflow catheter was inserted into the upper abdomen between the hepatic and diaphragmatic surface and an outflow catheter was placed at the pelvic floor. The perfusion solution was heated to 42.5 ± 0.5°C and infused into the peritoneal cavity at a rate of 10 mL/min through the inflow tube introduced from the automatic perfusion pump. The perfusion in the peritoneal cavity was stirred manually to make equal spatial distribution.

The temperature of the perfusion solution in peritoneal space was kept at 42.5 ± 0.5°C and monitored using a thermometer on real time. The total HIPEC time was 30 min, after which the perfusion solution in the abdominal cavity was removed. Twenty min before surgery, 100 mL of 0.9% sodium chloride solution with 1 g of ceftriaxone powder, 2 mL of 10% potassium chloride solution and 20 mL of 50% glucose solution was infused intravenously for rehydration, nutrition support and infection control in all the four groups of animals. Such treatment was continued for 3 d.

### Chemotherapy with PDOX and DOX after CRS + HIPEC

After CRS + HIPEC, the PDOX (n = 10) received systemic chemotherapy with PDOX 50.0 mg/kg (10.0 mg/kg and 3.0 mL/kg every 4 d for 5 cycles) on d 16, d 20, d 24, d 28, and d 32 after tumor cells inoculation respectively; the DOX (n = 10) received systemic chemotherapy with DOX 5.0 mg/kg (1.0 mg/kg and 3.0 mL/kg every 4 d for 5 cycles) at the same time; the Control and the HIPEC received 3.0 mL/kg 5% dextrose instead of chemotherapy drugs at the same time. All drugs were slowly injected i.v through the ear vein of rabbits. For each animal, 2.5 mg of dexamethasone was injected i.v 20 min before chemotherapy, and an additional 2 mL of 5% dextrose was injected i.v after PDOX or DOX injection to reduce local vascular damage.

### Animal observation and disease course monitoring

The general status of the animals was daily recorded in a standard form. For pathological studies, euthanasia was performed on the rabbits by overdose injection of 2% pentobarbital sodium through the ear vein. Post mortem pathological examinations included gross pathology such as tumor size and distributions; local tumor features of GC such as ulcer formation, intestinal obstruction and perforation; special features of PC such as bloody ascites, discrete or confluent tumor nodules on the peritoneum and omentum cake; metastases to major organs such as the livers and the lungs.

### Blood routine tests and biochemical test results

For laboratory studies, 3 mL of blood was harvested from ear vein on 2 d before tumor cells inoculation as the baseline (D-2), 2 d before CRS + HIPEC (D6), 2 d before chemotherapy (D14), and 4 d after completion of chemotherapy (D36). The samples were used for routine peripheral blood test, liver and kidney functions tests and detection of creatine kinase and creatine kinase mb isoenzyme.

### Histopathological studies under both optical microscope and electronic microscope

During the procedure of CRS, the resected omentum, tumor nodules, enlarged mesenteric lymph nodes were harvested for HE staining and observation under optical microscope. After post mortem pathological examinations, myocardial tissues of all the rabbits were harvested at the first time for the electronic microscope, and all the suspected organ metastasis tissues were sampled for routine histopathology study with sections stained by hematoxylin and eosin (HE stain).

### Statistical analyses

All data were integrated into a central database. The numerical data were directly recorded, and the category data were recorded into different categories. The primary endpoint was OS, and secondary endpoint was toxicity profile. Survival was analyzed on Kaplan-Meier survival plots and statistical significance was analyzed with SPSS software version 15.0 (SPSS Inc., IL, USA), using the log-rank test. The differences in blood routine and biochemistry among different groups were tested using nonparametric test at each time point. Categorized variables were compared by chi square test (*χ*^2^) or Fisher’s exact test. Two-sided *p* < 0.05 was considered to be statistically significant.

## Abbreviations

CTSB: Cathepsin B; PDOX: Peptide doxorubicin; DOX: Doxorubicin; PABC: Para-amino-benzyloxycarbonyl; PC: Peritoneal carcinomatosis; GC: Gastric cancer; CRS: Cytoreductive surgery; HIPEC: Hyperthermic intraperitoneal chemotherapy; OS: Overall survival; CI: Confidence intervals; ePCI: Experimental peritoneal carcinomatosis index; NMR: Nuclear magnetic resonance; MTD: Maximum tolerated dose; MCD: Minimum curative dose.

## Competing interests

PDOX has been filed for patent protection to the STATE INTELLECTUAL PROPERTY OFFICE OF P.R.C, with the application no: 201110300925.4 (inventors: Yan LI, Raymond A FIRESTONE, Ya-ping HONG and Li-hua SHAO).

## Authors’ contributions

Conceived and designed the experiments: YLI, LTANG. Performed the animal experiments: LT, Y-jZ, RD, H-lW. Chemical synthesis: Y-pH, J-gL, Y-cX, Analyzed the data: LT, RAF, Y-pH. Contributed reagents/materials/analysis tools: YLI, Y-pH, J-gL, Y-cX. Wrote the first draft of the manuscript: YLI, LT. Contributed to the writing of the manuscript: YL, LT. Y-pH. ICMJE criteria for authorship read and met: YL, LT. Agree with manuscript results and conclusions: YL, LT, Y-pH. Performed record linkage and selected data for analysis: LT. All authors read and approved the final manuscript.
